# Advanced atomic force microscopy-based techniques for nanoscale characterization of switching devices for emerging neuromorphic applications

**DOI:** 10.1186/s42649-021-00056-9

**Published:** 2021-05-26

**Authors:** Young-Min Kim, Jihye Lee, Deok-Jin Jeon, Si-Eun Oh, Jong-Souk Yeo

**Affiliations:** 1 School of Integrated Technology, Yonsei University, 85, Songdogwahak-ro, Yeonsu-gu, Incheon, 21983 Republic of Korea; 2 Yonsei Institute of Convergence Technology, Yonsei University, 85, Songdogwahak-ro, Yeonsu-gu, Incheon, 21983 Republic of Korea; 3 Nano Science and Engineering, Integrated Science and Engineering Division, Yonsei University, 85, Songdogwahak-ro, Yeonsu-gu, Incheon, 21983 Republic of Korea

**Keywords:** Selector, Conductive filaments (CFs), Conductive atomic force microscopy (C-AFM), Electrostatic force microscopy (EFM), Kelvin probe force microscopy (KPFM)

## Abstract

Neuromorphic systems require integrated structures with high-density memory and selector devices to avoid interference and recognition errors between neighboring memory cells. To improve the performance of a selector device, it is important to understand the characteristics of the switching process. As changes by switching cycle occur at local nanoscale areas, a high-resolution analysis method is needed to investigate this phenomenon. Atomic force microscopy (AFM) is used to analyze the local changes because it offers nanoscale detection with high-resolution capabilities. This review introduces various types of AFM such as conductive AFM (C-AFM), electrostatic force microscopy (EFM), and Kelvin probe force microscopy (KPFM) to study switching behaviors.

## Introduction

In modern society, technology with substantial data storage and high performance has become pivotal (Zhu et al. 2019). In 2008, Intel proposed a new type of nonvolatile memory device called storage class memory, which provides the advantages of scalability, low cost, and high performance (Burr et al. 2008; Pradel et al. 2011). Examples include resistive random access memory and phase change memory, offering nonvolatility and high-density characteristics (Lencer et al. 2008; Adinolfi et al. 2019). Recent advances in artificial intelligence increasingly require more efficient computing technologies, among which brain-inspired computing seems to be highly promising. To address current needs, it is necessary to develop materials, devices, and systems that closely mimic the human brain for neuromorphic applications. These systems require a paradigm shift to in-memory computing technologies in which memory and computing elements are integrated to serve the functionalities of neurons and synapses (Ielmini and Ambrogio 2019). To date, considerable research has been devoted to understand the correlations among switching mechanisms, device performance, and material systems in neuromorphic devices (Zhu et al. 2020).

To achieve high-density storage capacity, memory devices can be organized with array architectures such as cross-point arrays. However, existing arrays can have undesirable sneak currents to cells neighboring a target cell during operation. Introducing a selector with non-ohmic behavior can help solve this issue by designing one-selector/one-resistor (1S1R) configurations in cross-point arrays. The use of a separate two-terminal selector and memory cell enables independent control of each component (Song et al. 2018; Yoo et al. 2019). The key to understand switching mechanisms is studying the atomic behavior between a high resistive state (HRS), called an off-state, and a low resistivity state (LRS), called an on-state. The resistance change from the off-state to the on-state is called set and contrariwise is called reset, which occurs suddenly (Du et al. 2013). This can occur at a specific voltage called the threshold voltage (V_th_), which induces undefined changes in switching materials. Therefore, with innovative memory devices in high demand, the role of selector devices and a thorough understanding of the switching mechanism are critical to obtain high-performance devices.

Xu et al. (2014) reported that the switching mechanisms in memristive devices produce conductive filaments (CFs) in the switching layer (SL). CF-forming mechanisms such as filaments form along with oxygen vacancies (Ju et al. 2017) and electrode diffusion (Xu et al. 2014). Other theories have recently been published to elucidate the switching behavior such as field-induced electron hopping transport (Ielmini and Zhang 2007) and metastable metavalent bond formation (Noé et al. 2020; Raty and Noe 2020). According to research on the switching mechanisms within SLs, local changes occur within a small area of ~ 100 nm^2^ in the layer (Lanza 2014; Lanza et al. 2017). However, it is difficult to determine where the switching phenomenon precisely occurs with global switching using a probe station. This is because it collects electronic signals for whole area under active electrode while measuring the I-V curve (Lanza 2014). Therefore, analytical tools that can characterize the nanoscale changes are needed to observe high-resolution switching. Transmission electron microscopy (TEM) is a powerful tool that is used to analyze local characteristics with a high nanoscale resolution. However, samples should be thin enough for electrons to transmit through, which can induce unexpected thermal effects that affect switching behavior. Samples also require high vacuum conditions to operate as they use an electron source. For these reasons, atomic force microscopy (AFM) is a suitable tool to study local changes among nanoscale analysis approaches. As it has a sharp detecting tip of 10–20 nm, AFM can characterize local changes in tens of nanometer scale. AFM does not require strict sample conditions and high vacuum; thus, it can be used with relative ease (Lee and Hwang 2011).

**Fig. 1:**
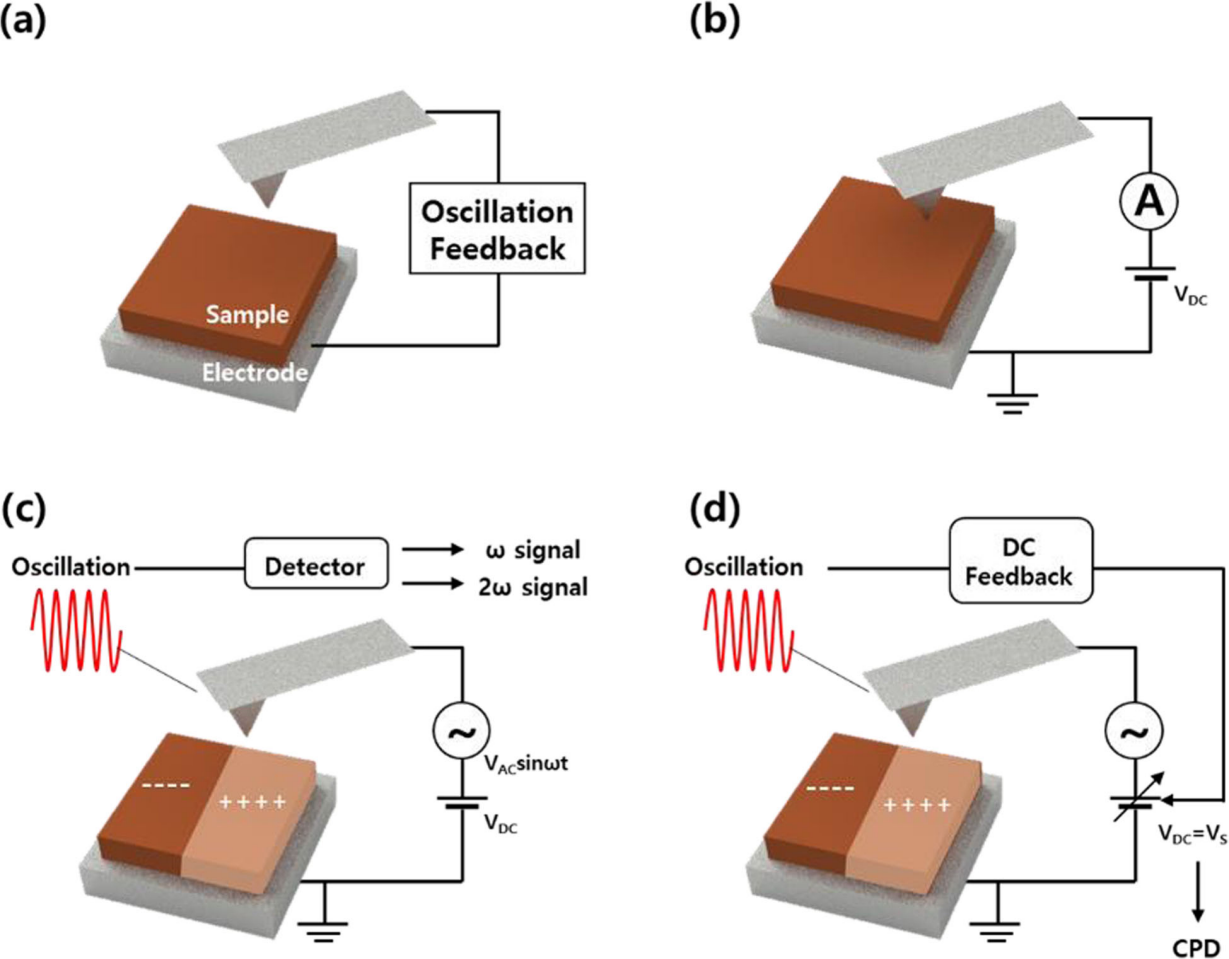
Schematics of **a** conventional non-contact atomic force microscopy (NC-AFM), **b** conductive atomic force microscopy (C-AFM), **c** electrostatic force microscopy (EFM), and **d** Kelvin probe force microscopy (KPFM)

This review provides an overview of recent technological developments that analyze switching behavior using advanced AFM-based techniques such as conductive atomic force microscopy (C-AFM), electrostatic force microscopy (EFM), and Kelvin probe force microscopy (KPFM) compared to conventional non-contact atomic force microscopy (NC-AFM). Figure 1 shows schematics of NC-AFM (Fig. 1a), C-AFM (Fig. 1b), EFM (Fig. 1c), and KPFM (Fig. 1d). Information that can be assessed through AFM-based tools includes topography, electrical conduction, current mapping images, surface potential, and work functions of materials but can be extended beyond these capabilities. Each method offers unique and powerful features that demonstrate switching mechanisms based on local area analysis.

### Conductive atomic force microscopy

In C-AFM, conductive materials such as metal, conductive diamond are coated to the AFM tips in order to measure the current value. Tips with nanoscale dimensions can read localized electrical information when a bias is applied to samples through contact scanning tips. High-resolution spatial current mapping data measured by C-AFM shows the differences in the surface’s electrical properties according to whether the switching operation has occurred in nanoscale (Waser and Aono 2007). However, the range of current value of C-AFM is fA ~ μA, which is lower than conventional I-V measurement tools (e.g., probe stations). In C-AFM, the current value can be easily saturated when conductive materials are measured. Thus, each material requires an appropriate gain to be set to analyze the switching behavior.

### Analysis of 2D local domain morphology of switching film surfaces by electrical bias

**Fig. 2:**
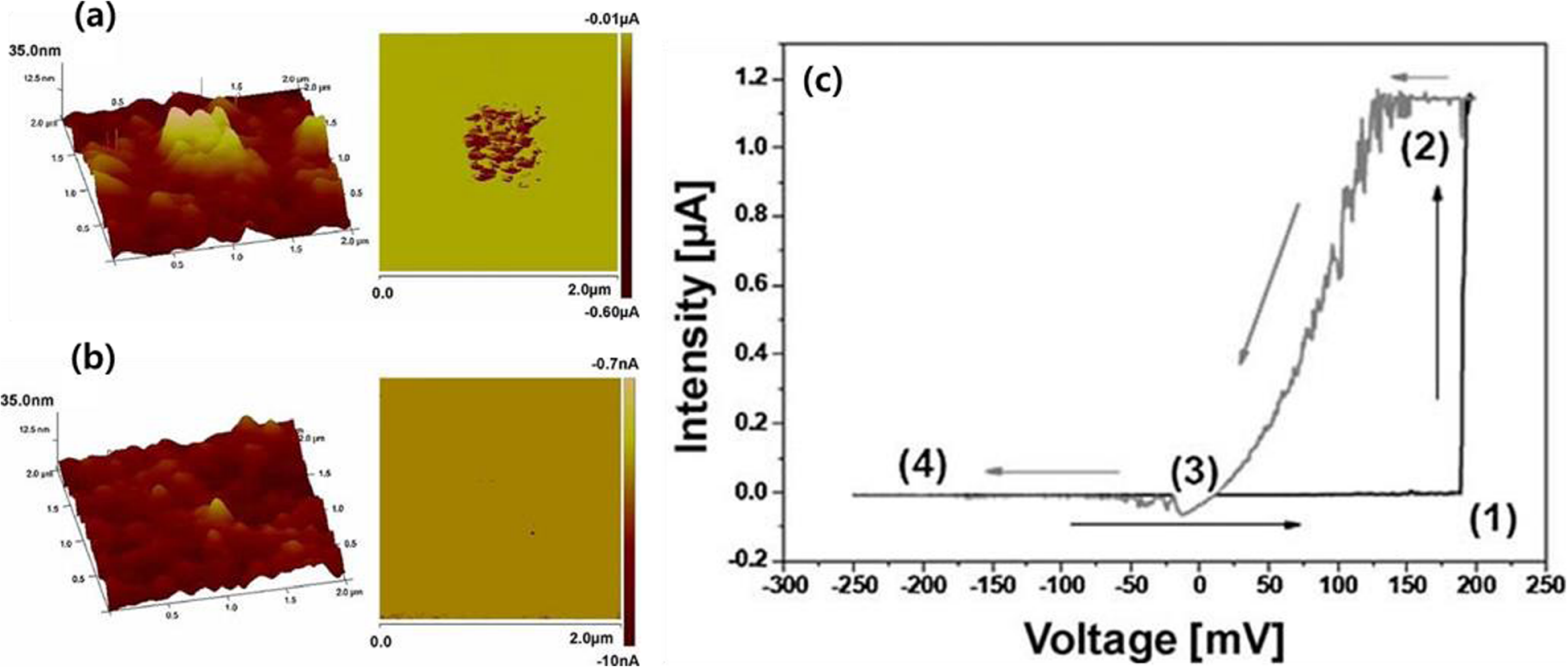
Surface morphology changes in the switching layer when voltage is applied to a film via C-AFM. **a** The surface of an Ag/Ge_0.25_Se_0.75_ film applied with + 200 mV (V_wr_) shows images of local morphology (left) and 2D current mapping (right). **b** The surface of an Ag/Ge_0.25_Se_0.75_ film applied with − 250 mV (V_er_) shows images of local morphology (left) and 2D current mapping (right). **c** I-V curves: (1) writing step, (2) on-state, (3) erasing step, and (4) off-state. Reprinted from Pradel et al. (2011) (*Phys. Status Solidi A* 208, 2303–2308) with *Physica Status Solidi A*’s permission

As previously mentioned, C-AFM utilizes a nanometer-scale tip that is used to characterize precise differences in the topography of the switching area that is selectively biased (Lanza et al. 2017; O’shea et al. 1995). Yoo et al. (2008) demonstrated the measurement of CF formation by scanning NiO thin films with C-AFM. Some regions with higher current levels were characterized with nanoscale resolutions as the switching bias was applied to the specific surface SL region. These spots were randomly present on the film’s surface, and their number varied during each switching test. This variation in conducting path characteristics can lead to nonuniformity in overall switching properties. Moreover, other researchers confirmed that switching operation induces local changes that do not have the same characteristics. Bosse et al. (2014) compared current ratios at different spots from the area under the same switching cycle. Each spot had different current values that were likely induced by slight variations such as local composition and structural differences on the surface. Analyzing the heterogeneity of a film’s surface is necessary to evaluate the performance of switching devices based on film. It is also important to analyze a specific conducting region because each conducting path can be correlated to the switching properties. Pradel et al. (2011) demonstrated bipolar resistance switching in chalcogenide materials using C-AFM. Figure 2 shows topographical and current images after the switching cycle. The roughness and current value results at the write voltage (V_wr_) (Fig. 2a) and erase voltage (V_er_) (Fig. 2b) obviously differ and are shown as I-V curves (Fig. 2c). Changes in the morphology and current value occur at + 200 mV (V_wr_) and disappear at − 250 mV (V_er_). The surface roughness and conductivity are attributed CF formation by Ag^+^ migration as cations are attracted to the tip of the C-AFM because there were no signs of switching when the experiment was conducted without Ag from the electrode. However, it was unclear whether CF can also form with symmetric structures between electrodes. Thus, the results of a surface analysis of the specimen cannot be correlated to the inner layer. As this analysis can be limited to accurately characterize CFs, it is necessary to develop other techniques that can directly observe the inside of the SL.

### Direct observation of conducting filaments inside switching film via 3D tomography analysis

**Fig. 3:**
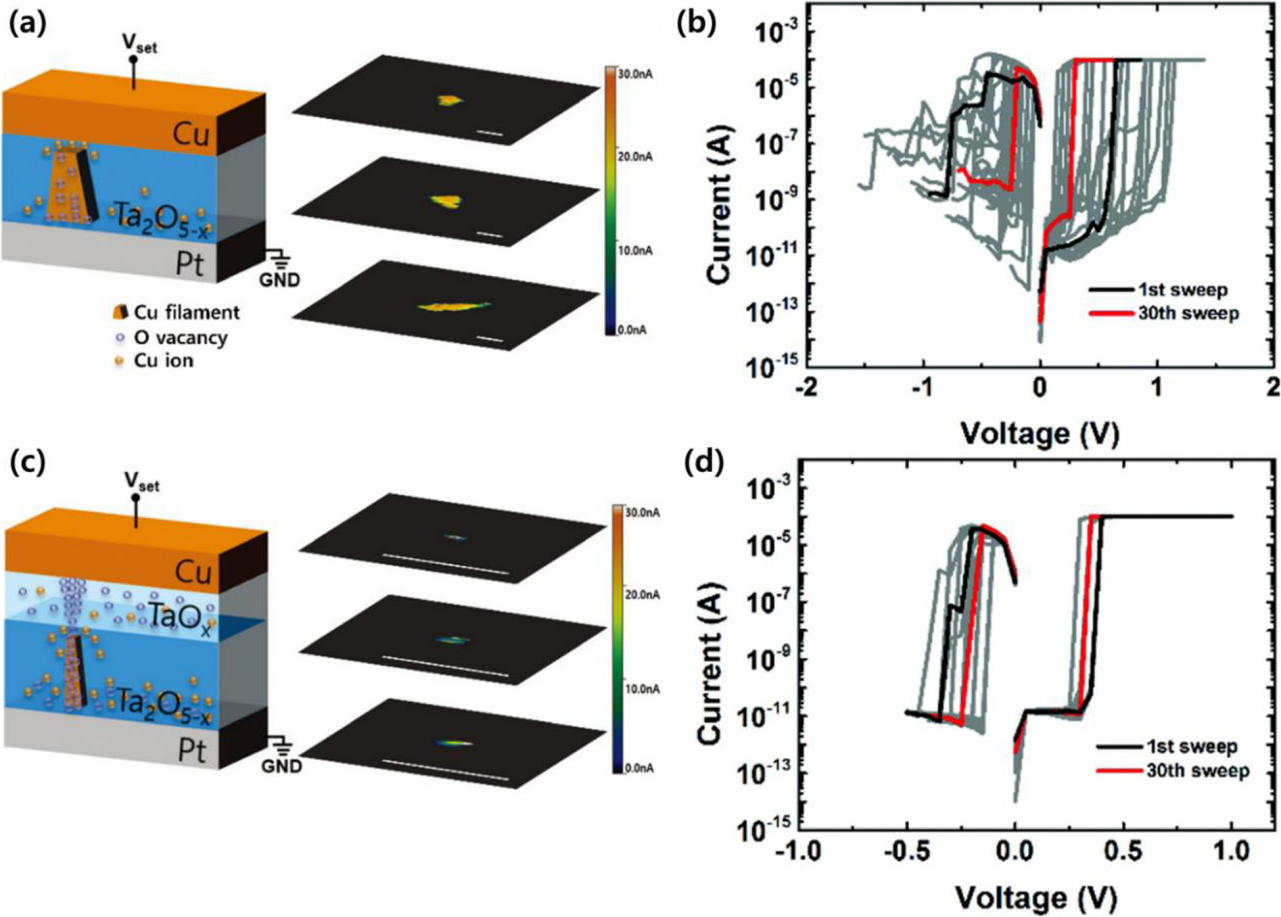
Schematic illustrations of the set process and 3D current mapping images of conductive filaments through C-AFM depth profile analysis and I-V curves. **a** Single-layer resistive switching devices (left) and current mapping images of the Ta_2_O_5-x_ layer (right) during the set process, scale bars: 100 nm. **b** I-V curve after the 1st and 30th sweeps. **c** Bi-layer resistive switching devices (left) and current mapping images of the TaO_x_ and Ta_2_O_5-x_ layers (right) during the set process, scale bars: 100 nm. **d** I-V curve after the 1st and 30th sweeps. Reprinted from Ju et al. (2017) (*Nanoscale* 9, 8373–8379) with *Nanoscale*’s permission

To clearly understand the formation of CFs, it is better to directly confirm from inside the switching film. C-AFM can detect changes before and after switching operations through 3D imaging (Ju et al. 2017; Celano et al. 2014). Celano et al. (2014) first reported 3D tomography that was applied to verify CFs formed by switching cycles. They removed the top electrode and repeated the slice and scan process on the SL using conventional conductive C-AFM with a diamond tip. The shapes of the CFs were conical, and the cross-sectional area narrowed as it was closer to the inert electrode. Analyzing the structural and morphological properties is helpful to understand the switching process. Therefore, characterization of CFs is a necessary step to improve the performance of selector devices that operate based on CF formation. In research relevant to improving resistive switching devices, changing the composition of SL can control the width of the CFs, thus affecting their switching properties (Ju et al. 2017). As many oxygen vacancies in the inserted layer induce the formation of oxygen ions in the SL causing the CFs to grow toward the vertical direction predominant in the SL, the widths of the CFs are controlled by introducing a functional oxide layer (TaO_x_) to produce a bi-layer (Ta_2_O_5-x_/TaO_x_). In single-layer devices, CFs consisting of Cu provide larger widths than bi-layer devices due to uncontrolled growth of the filament (Fig. 3a). The residual filament remains after the switching process in the SL and the bias to turn on the switching device progressively declines as the switching cycle increases in the I-V curves (Fig. 3b). Conversely, there is no remaining residual filament in the SL of bi-layer devices after the switching cycle (Fig. 3c), and relatively consistent switching performance is demonstrated regardless of the number of switching cycles in the I-V curves (Fig. 3d).

C-AFM enables the measurement of electrical properties as proof of the switching behavior and has a spatially high-resolution detecting capability due to the contact mode-based scanning process. Although the C-AFM-based analysis is a good approach to study switching behavior, localized stimulation at small nanoscale areas may impose significant local stresses such as large thermal and electrical effects. This can induce physical damage in the samples as reported in prior research (Polspoel and Vandervorst 2007; Gołek et al. 2014). The high sensitivity of C-AFM may cause poor reproducibility depending on ambient environment such as humidity since it affects water meniscus between the tip and the sample. Because contact force is affected by relative humidity (Thundat et al. 1993), it is important to keep ambient conditions as consistent as possible to get reliable data.

### Electrostatic force microscopy and kelvin probe force microscopy

Aside from C-AFM, EFM can be used to detect variations in electric charges during the switching process. EFM measures electrostatic interactions between a tip and a sample in a noncontact state. Unexpected effects such as physical damages caused by contact can be excluded when analyzing switching behavior. Generally, the dual scan path method is applied to avoid interference between electrostatic forces and van der Waals forces, which are different types of forces according to the tip sample distance (Yan and Bernstein 2007). The first scan reads the topographical information based on van der Waals forces near surface of the sample and the second scan reads electrostatic force-based interactions at specific heights along the line recorded during the first step. The electrostatic force is expressed by Eq. (1) (Cherniavskaya et al. 2003):

1 $$ {F}_{EFM}={F}_{cap}+{F}_{coul}=\frac{1 dC}{2 dz}{V}_{tot}^2+{E}_Z{Q}_{tip} $$

where *F*_*EFM*_ is the total electrostatic force that is composed of a capacitive force (*F*_*cap*_) and Coulombic force (*F*_*coul*_), *dC/dz* is the derivative of the empty tip sample capacitance according to the tip sample distance, *z*, *V*_*tot*_ is the total voltage, *E*_*z*_ is the contribution of the electric field, and *Q*_*tip*_ is the sum of the charge on the tip. *F*_*cap*_ is proportional to the square of the total voltage (*V*_*tot*_), which consists of three terms: DC bias (*V*_*DC*_), AC bias with a *ω* angular frequency (*V*_*AC*_ sin*ωt*), and the voltage difference (V_S_) between the tip and sample. The 2*ω* part can be acquired from trigonometric function rules. Therefore, there are two types of electrostatic force functions, *F*(*ω*) and *F* (2*ω*), which are affected by the electrostatic force that forms due to charge accumulation on the surface, respectively. This induces a mechanical phase shift, *ΔΦ*, of the tip oscillation during the scan process as described by Eq. (2) (Arinero et al. 2013):

2 $$ \Delta  \varPhi \cong -\frac{Q_n}{k_n}F{\prime}_{EFM} $$

where *Q*_*n*_ is the quality factor, *k*_*n*_ is the cantilever’s dynamic stiffness, and *F’*_*EFM*_ is the differential electrostatic force. The results of EFM scans appear as phase shifts depending on the oscillation amplitude of the cantilever according to the interactions between the tip and sample at *ω* and 2*ω* frequencies. KPFM is also based on the same interaction force and the corresponding equations from EFM measurements, but there is a *V*_*DC*_ feedback that makes the *ω* signal zero value (*F*(*ω*) = 0) in the KPFM. Using this feedback algorithm, the contact potential difference (CPD) is quantitatively measured, which cannot be achieved by EFM.

### Analysis of the surface charge state after the switching cycle using EFM

**Fig. 4:**
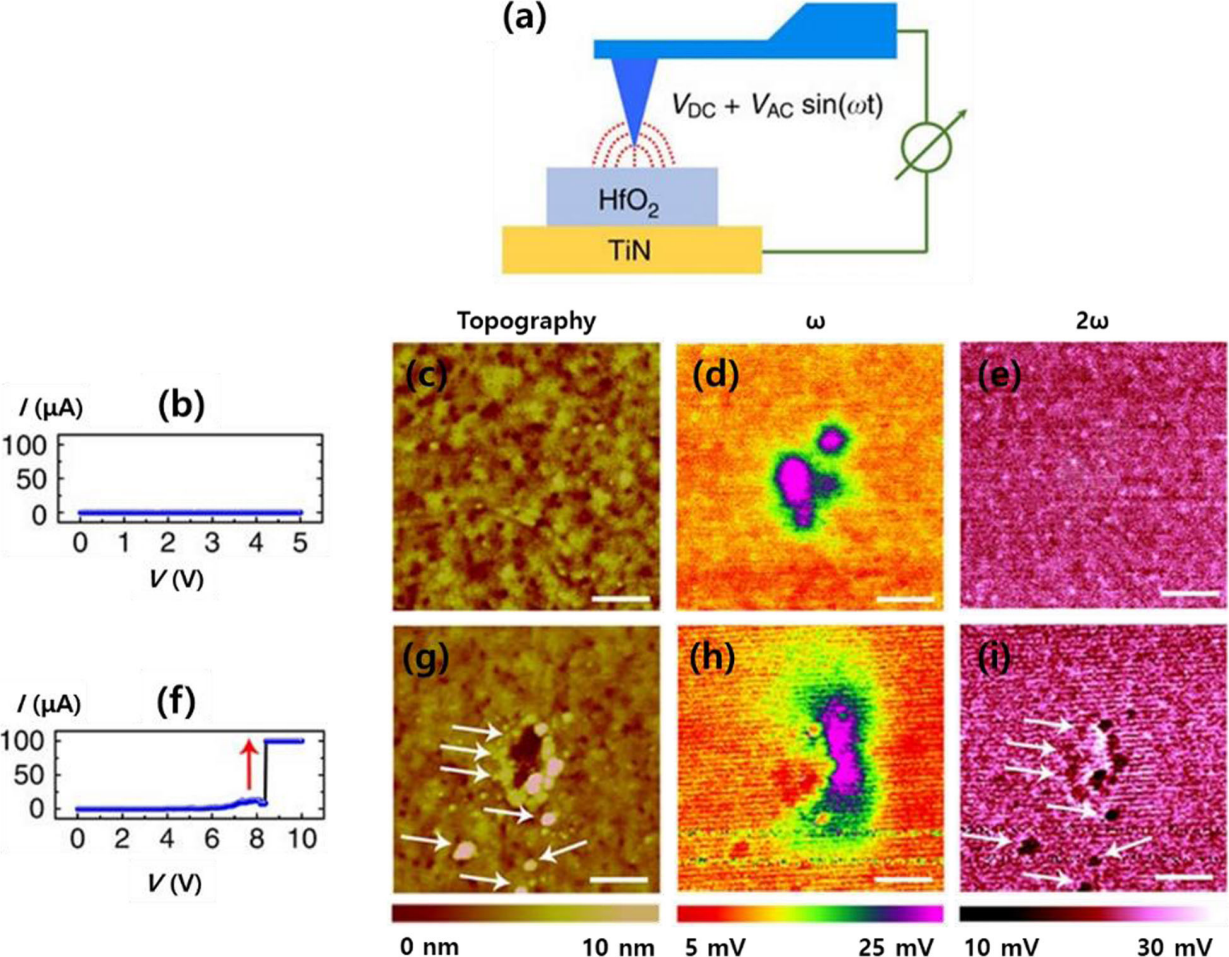
Probing HfO_2_ film during the switching process using EFM. **a** Schematic of electrostatic force microscopy (EFM) measurements of an HfO_2_/TiN system. **b-e** Electrical properties (**b**), topography (**c**), ω (**d**), and 2ω (**e**) results up to 5 V during voltage sweep. **f-i** Electrical property (**f**), topography (**g**), ω (**h**), and 2ω (**i**) results up to 10 V during voltage sweep. (Scale bar in the images, 4 μm). Reprinted from Yang et al. (2017) (*Nat*. *Commun*. 8:15173) with *Nature Communication*’s permission

EFM is used to study ion migration and accumulation due to its specific sensitivity to the charge of ions (Yang and Huang 2018). On the basis of these characteristics of EFM measurement, the effects of oxygen ion behavior were investigated through the switching process in HfO_2_-based memristive systems (Yang et al. 2017). The authors reported that the movement of oxygen ions during the switching process in the off-to-on state in HfO_2_ films can be confirmed by *ω* and 2*ω* factors relevant to charge accumulation and capacitance between the tip and sample surface, respectively. During EFM scanning (Fig. 4a), the samples are swept up to 5 V and 10 V, which demonstrates different electrical properties (Fig. 4b and f), topography (Fig. 4c and g), and images of *ω* (Fig. 4d and h) and 2*ω* (Fig. 4e and i) signal scans, respectively. In *ω* signal images, bias-applied regions show changes in electrostatic forces depending on the charge accumulation by oxygen anions. The reason why charge accumulates via oxygen anions, not electrons, is demonstrated by the charge remaining time of more 1 h after removing bias. The charge accumulation is shown in the 5 V and 10 V samples. However, in the 2*ω* signal image and morphology, the switching behavior involves structural deformation and changes in capacitance. The relationship between the two results can be explained as follows. If the oxygen anion-accumulated regions are biased by over-the-threshold voltage, oxygen anions are oxidized to emanate oxygen gas, causing structural deformation and local non-stoichiometry in the HfO_2_ films, which affect the capacitance properties. These results expand the understanding of switching mechanisms on oxide-based memristive devices by showing the changes on the film’s surface. In addition to this research on the filamentary switching phenomenon, there is a study related to non-filamentary switching device by sulfur vacancy migration (Sangwan et al. 2015). During switching process of the memristor device, the researchers identified abrupt phase changes by accumulation of sulfur vacancies at grain boundary in the MoS_2_ monolayer. However, as previously mentioned, comparisons based on EFM are limited as they only provide a qualitative interpretation.

### Quantitative analysis of electrostatic interactions using KPFM

**Fig. 5:**
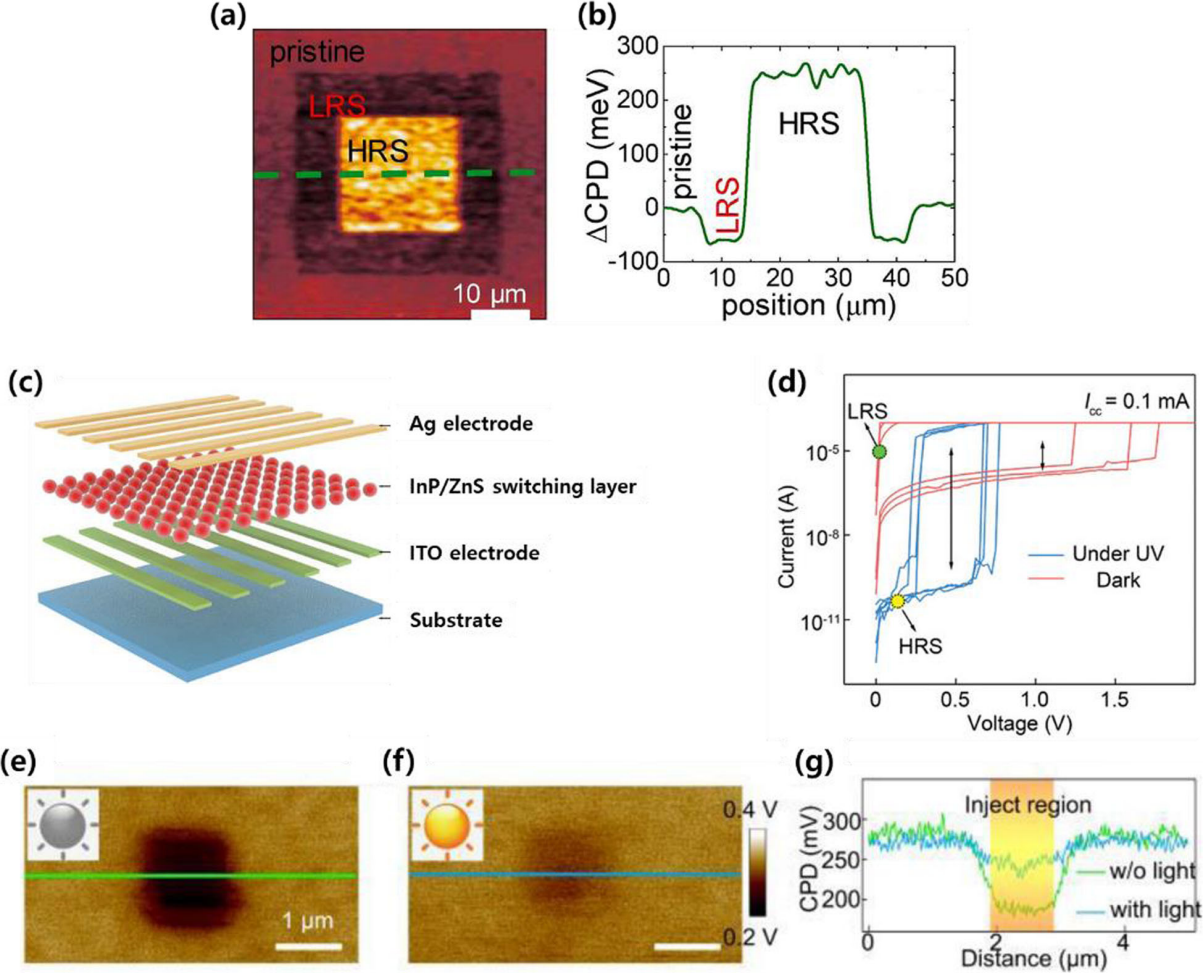
CPD mapping of switching films using KPFM. **a-b** Measurements of strontium iridate-based thin films. **a** CPD map measured under pristine (0 V), LRS (4.5 V), and HRS (− 6 V) conditions. **b** CPD profile along the green dashed line in (**a**) (Fuentes et al. 2020). **c-g** Measurements of InP/ZnS quantum dot-based films. **c** Schematic structure of InP/ZnS quantum dot-based switching devices. **d** I-V curve that shows changes in switching types under UV irradiation and dark states. **e-g** CPD maps measured under dark (**e**) and UV light states (**f**). **g** CPD profile comparison between the green line in (**e**) and the blue line in (**f**) (Wang et al. 2020). Reprinted from Fuentes et al. (2020) and Wang et al. (2020) (*J*. *Magn*. *Magn*. *Mater*. 501, 166,419 and *Adv*. *Funct*. *Mater*. 30, 1,909,114) with *Journal of Magnetism and Magnetic Materials* and *Advanced Functional Materials’* permission

Another form of EFM that is a more advanced version is Kelvin probe force microscopy (KPFM). KPFM enables the quantitative measurement of the local contact potential difference (CPD) between the microscopy tip and the sample and mapping the work function or the potential of the sample’s surface with a high resolution (Du et al. 2013; Maragliano et al. 2014). As this measurement is based on the noncontact mode, it also helps to avoid extra effects by the Schottky barrier that can occur at the tip sample junction (Kumar and Som 2015). The resistive switching process is investigated via CPD mapping using KPFM (Fuentes et al. 2020). Strontium iridate (Sr_n + 1_Ir_n_O_3n + 1_)-based thin films have different electric conductivities according to the composition ratio and show resistive switching properties in Sr_2_IrO_4_ compositions. Their pristine set (LRS)-reset (HRS) states are characterized through KPFM measurement at a low voltage of 1 V, a positive voltage of + 4.5 V, and a negative voltage of − 6 V (Fig. 5a), respectively. Each state’s quantitative values are detected as the CPD between the tip and sample (Fig. 5b). In these experiments, the results show changes in resistance states in nonvolatile resistive switching after the switching cycle. The following describes a recent study on changes in switching types by analyzing peripheral charges of CFs inside films. Wang et al. (2020) reported an analysis using KPFM on instant changes between threshold switching and resistive switching during UV irradiation of films with InP/ZnS quantum dot-based memristive devices. There is a quantum dot-based switching layer between Ag and ITO electrodes (Fig. 5c). Its switching characteristics change depending on the presence or absence of UV light irradiation (Fig. 5d). Using the KPFM results, the researchers compared the two regions of 1 μm^2^ in area where a charge was injected to cause the switching operation. One film is under a dark condition while the other is under a UV-irradiated condition (Fig. 5e and f). There is a distinct difference of approximately 100 mV in the CPD value between the charge-injected area and non-injected area under the dark condition. In this experiment, CFs form by Ag ions migrating from the Ag electrode, resulting in non-volatile properties. This leads to a relatively larger CPD difference between the charge injected area and non-injected area. However, there is a 30 mV difference in the CPD value under UV-irradiated conditions (Fig. 5f). As Ag CF tends to undergo oxidation reduction or redox reactions, an environment with sufficient hole concentrations induced by UV irradiation can allow the filament to dissociate enough to an off-state (initial state). As a result, the switching characteristics can be altered between volatile and nonvolatile switching depending on whether the film is irradiated with UV light, and their properties can be easily measured using KPFM (Fig. 5g).

## Conclusion

Switching behavior occurring in local areas can be observed using advanced AFM-based techniques. The C-AFM, one type of advanced AFM tool, can read electrical and geometric information on the surface. Changes inside the SL can be directly observed through 3D tomography. Using EFM, another type of AFM, switching processes can be studied based on the electrostatic interactions without sample damage. The changes in charge accumulation and capacitance are qualitatively observed after the switching cycle. These results can be quantified by calculating the CPD using KPFM. However, AFM-based analyses require additional research to accurately understand the switching mechanisms.

A real-time analysis method was recently demonstrated using EFM (Kajimoto et al. 2020). In this study, the charge migration on a polymer film was confirmed on a micro-second time scale. The results can be extended to observe progressive changes induced by switching in higher time resolutions. Savage et al. (2012) measured the tunneling current and quantum conductance using a super continuum laser and C-AFM. As shown in this study, existing AFM-based tools can be integrated with other measurement designs to develop novel analytical techniques to elucidate switching mechanisms. Extended understanding based on the nanoscale characterization of switching devices using advanced AFM-based techniques can facilitate the optimization and performance improvement of emerging devices for in-memory computing and neuromorphic applications.

## Data Availability

Not applicable.

## Abbreviations

AFM: atomic force microscopy
NC-AFM: non-contact atomic force microscopy
C-AFM: conductive atomic force microscopy
EFM: electrostatic force microscopy
KPFM: Kelvin probe force microscopy
1S1R: one selector-one resistance
HRS: high-resistance state
LRS: low-resistance state
V_th_: threshold voltage
CFs: conductive filaments
SL: switching layer
V_wr_: write voltage
V_er_: erase voltage
CPD: contact potential difference

## References

[CR1] Adinolfi V, Cheng L, Laudato M, Clarke RC, Narasimhan VK, Balatti S, Hoang S, Littau KA (2019). Composition-controlled atomic layer deposition of phase-change memories and ovonic threshold switches with high performance. ACS Nano.

[CR2] Arinero R, Trasobares J, Girard P, Ramonda M, Clément N (2013). Temperature and damping effects on the frequency dependence of electrostatic force microscopy force gradients. J. Appl. Phys..

[CR3] Bosse JL, Grishin I, Gyu Choi Y, Cheong B-k, Lee S, Kolosov OV, Huey BD (2014). Nanosecond switching in GeSe phase change memory films by atomic force microscopy. Appl. Phys. Lett..

[CR4] Burr GW, Kurdi BN, Scott JC, Lam CH, Gopalakrishnan K, Shenoy RS (2008). Overview of candidate device technologies for storage-class memory. IBM J. Res. Dev..

[CR5] Celano U, Goux L, Belmonte A, Opsomer K, Franquet A, Schulze A, Detavernier C, Richard O, Bender H, Jurczak M (2014). Three-dimensional observation of the conductive filament in nanoscaled resistive memory devices. Nano Lett..

[CR6] Cherniavskaya O, Chen L, Weng V, Yuditsky L, Brus LE (2003). Quantitative noncontact electrostatic force imaging of nanocrystal polarizability. J. Phys. Chem. B..

[CR7] Du Y, Kumar A, Pan H, Zeng K, Wang S, Yang P, Wee ATS (2013). The resistive switching in TiO2 films studied by conductive atomic force microscopy and kelvin probe force microscopy. AIP Adv..

[CR8] Fuentes V, Vasić B, Konstantinović Z, Martínez B, Balcells L, Pomar A (2020). Resistive switching in strontium iridate based thin films. J. Magn. Magn. Mater..

[CR9] Gołek F, Mazur P, Ryszka Z, Zuber S (2014). AFM image artifacts. Appl. Surf. Sci..

[CR10] Ielmini D, Ambrogio S (2019). Emerging neuromorphic devices. Nanotechnol..

[CR11] Ielmini D, Zhang Y (2007). Analytical model for subthreshold conduction and threshold switching in chalcogenide-based memory devices. J. Appl. Phys..

[CR12] Ju JH, Jang SK, Son H, Park J-H, Lee S (2017). High performance bi-layer atomic switching devices. Nanoscale.

[CR13] Kajimoto K, Araki K, Usami Y, Ohoyama H, Matsumoto T (2020). Visualization of charge migration in conductive polymers via time-resolved electrostatic force microscopy. J. Phys. Chem. A.

[CR14] Kumar M, Som T (2015). Structural defect-dependent resistive switching in cu-O/Si studied by kelvin probe force microscopy and conductive atomic force microscopy. Nanotechnology.

[CR15] Lanza M (2014). A review on resistive switching in high-k dielectrics: A nanoscale point of view using conductive atomic force microscope. Materials.

[CR16] Lanza M, Celano U, Miao F (2017). Nanoscale characterization of resistive switching using advanced conductive atomic force microscopy based setups. J. Electroceram..

[CR17] Lee MH, Hwang CS (2011). Resistive switching memory: Observations with scanning probe microscopy. Nanoscale.

[CR18] Lencer D, Salinga M, Grabowski B, Hickel T, Neugebauer J, Wuttig M (2008). A map for phase-change materials. Nat. Mater..

[CR19] Maragliano C, Lilliu S, Dahlem M, Chiesa M, Souier T, Stefancich M (2014). Quantifying charge carrier concentration in ZnO thin films by scanning kelvin probe microscopy. Sci. Rep..

[CR20] Noé P, Verdy A, d’Acapito F, Dory J-B, Bernard M, Navarro G, Jager J-B, Gaudin J, Raty J-Y (2020). Toward ultimate nonvolatile resistive memories: The mechanism behind ovonic threshold switching revealed. Sci. Adv..

[CR21] O’shea S, Atta R, Murrell M, Welland M (1995). Conducting atomic force microscopy study of silicon dioxide breakdown. J. Vac. Sci. Technol. B.

[CR22] Polspoel W, Vandervorst W (2007). Evaluation of trap creation and charging in thin SiO2 using both SCM and C-AFM. Microelectron. Eng..

[CR23] Pradel A, Frolet N, Ramonda M, Piarristeguy A, Ribes M (2011). Bipolar resistance switching in chalcogenide materials. Phys. Status Solidi A.

[CR24] J. Y. Raty, P. Noe, Ovonic Threshold Switching in Se-Rich GexSe1-x Glasses from an Atomistic Point of View: The Crucial Role of the Metavalent Bonding Mechanism. Phys. Status. Solidi-R. 1900581 (2020)

[CR25] Sangwan VK, Jariwala D, Kim IS, Chen K-S, Marks TJ, Lauhon LJ, Hersam MC (2015). Gate-tunable memristive phenomena mediated by grain boundaries in single-layer MoS 2. Nat. Nanotechnol..

[CR26] Savage KJ, Hawkeye MM, Esteban R, Borisov AG, Aizpurua J, Baumberg JJ (2012). Revealing the quantum regime in tunnelling plasmonics. Nature.

[CR27] Song B, Xu H, Liu S, Liu H, Li Q (2018). Threshold switching behavior of Ag-SiTe-based selector device and annealing effect on its characteristics. IEEE J. Electron. Devices Soc..

[CR28] Thundat T, Zheng X-Y, Chen G, Warmack R (1993). Role of relative humidity in atomic force microscopy imaging. Surf. Sci. Lett..

[CR29] Wang J, Lv Z, Xing X, Li X, Wang Y, Chen M, Pang G, Qian F, Zhou Y, Han ST (2020). Optically modulated threshold switching in Core–Shell quantum dot based Memristive device. Adv. Funct. Mater..

[CR30] Waser R, Aono M (2007). Nanoionics-based resistive switching memories. Nat. Mater..

[CR31] Xu X, Lv H, Liu H, Gong T, Wang G, Zhang M, Li Y, Liu Q, Long S, Liu M (2014). Superior retention of low-resistance state in conductive bridge random access memory with single filament formation. IEEE Electron. Device Lett..

[CR32] Yan M, Bernstein GH (2007). A quantitative method for dual-pass electrostatic force microscopy phase measurements. Surf. Interface Anal..

[CR33] Yang Y, Huang R (2018). Probing memristive switching in nanoionic devices. Nat. Electron..

[CR34] Yang Y, Zhang X, Qin L, Zeng Q, Qiu X, Huang R (2017). Probing nanoscale oxygen ion motion in memristive systems. Nat. Commun..

[CR35] Yoo I, Kang B, Park Y, Lee M, Park Y (2008). Interpretation of nanoscale conducting paths and their control in nickel oxide (NiO) thin films. Appl. Phys. Lett..

[CR36] Yoo J, Kim SH, Chekol SA, Park J, Sung C, Song J, Lee D, Hwang H (2019). 3D stackable and scalable binary ovonic threshold switch devices with excellent thermal stability and low leakage current for high-density cross-point memory applications. Adv. Electron. Mater..

[CR37] Zhu J, Zhang T, Yang Y, Huang R (2020). A comprehensive review on emerging artificial neuromorphic devices. Appl. Phys. Rev..

[CR38] Zhu M, Ren K, Song Z (2019). Ovonic threshold switching selectors for three-dimensional stackable phase-change memory. MRS Bull..

